# Anaesthesia management of a patient with Bethlem Myopathy for elective tonsillectomy: a case report

**DOI:** 10.1186/s12871-024-02539-0

**Published:** 2024-05-10

**Authors:** Conor McGarrigle, Launcelot McGrath, Ehtesham Khan

**Affiliations:** Our Lady of Lourdes Hospital, Drogheda, Ireland

**Keywords:** Bethlem Myopathy, Hereditary muscular disorder, Collagen VI-related myopathy

## Abstract

**Background:**

Bethlem Myopathy is a collagen VI-related myopathy presenting as a rare hereditary muscular disorder with progressive muscular weakness and joint contractures. Despite its milder clinical course relative to other myopathies, anaesthetic management can be challenging. High arched palates and fixed flexion deformities may contribute to a difficult airway. A progressive decline in pulmonary function can present later into adulthood. This respiratory decline can carry secondary cardiovascular consequences due to the progressive nature of restrictive lung disease, including right sided heart disease and pulmonary hypertension. We describe a case of a male patient with Bethlem Myopathy undergoing anaesthesia, to contribute to the limited body of literature on this condition and enhance awareness and guidance amongst anaesthesiologists on approaching patients with this condition. This is the first case report within the literature of its kind.

**Case presentation:**

This case details a 33-year-old male with Bethlem Myopathy undergoing tonsillectomy. Diagnosed in childhood following developmental delays, the patient had no prior anaesthetic exposure and no family history of anaesthetic complications. Anaesthetic induction was achieved without complications, avoiding depolarizing muscle relaxants and careful airway management. Extreme care was taken in patient positioning to prevent complications. The surgery proceeded without incident and muscle paralysis was reversed with Suggammadex, resulting in no adverse post-operative respiratory complications. The patient was discharged on the first post-operative day without any respiratory or cardiovascular compromise.

**Conclusions:**

Bethlem Myopathy, while often exhibiting a mild clinical course, can present anaesthetic challenges. Awareness of potential complications including a difficult airway, cardiovascular and respiratory implications as well as the need for specialised monitoring and positioning is crucial to ensure a safe peri-operative course.

## Background

Bethlem’s Myopathy is a rare hereditary muscular disorder characterised by progressive muscular weakness and joint contractures. It typically presents in early childhood, with a delay in reaching motor milestones. A Heterogenic autosomal dominant disorder, first described by Bethlem in 1976, it is a subtype of collagen VI related myopathies, caused by mutations in the genes responsible for collagen type VI synthesis [1, 2]. Collagen VI plays a pivotal role in maintaining the structural integrity and stability of the extracellular matrix, with its residual functionality determining the clinical severity of the disorder [3, 4]. It is considered a more benign variant of Collagen VI myopathy, with Ullrich myopathy positioned on the severe end of the clinical spectrum.

We describe a case of a patient with Bethlem Myopathy undergoing anaesthesia, to contribute to the limited body of literature on this condition and enhance awareness and guidance amongst anaesthesiologists on approaching patients with this condition. This is the first case report within the literature of its kind.

## Case presentation

A 33-year-old male presented electively for a tonsillectomy under a general anaesthetic. A diagnosis of Bethlem’s Myopathy was made by a paediatric specialist early in the patient’s childhood due to concerns about a delay in developmental motor milestones in his infancy. The diagnosis was confirmed through a muscle biopsy as a child. A family history revealed similar issues among relatives, but no previous formal diagnoses were made.

In preparation for the tonsillectomy, the patient was reviewed in the anaesthetic preassessment clinic. He had no prior anaesthetic exposure and there was no family history of any anaesthesia related complications, including malignant hyperthermia.

His past medical history was negative for any cardiovascular or respiratory pathology. Given his myopathic disorder, he attended biannual surveilling transthoracic echocardiograms, under the care of a cardiologist. A recent echocardiogram demonstrated a dilated ascending aorta within the upper limits of normal range and was managed conservatively. There were no valvular or cardiac chamber abnormalities or evidence of cardiomyopathy. He reported good exercise tolerance, was ambulatory and experienced no respiratory limitations or shortness of breath within his daily activities. Additionally, he receives annual eye examinations under the care of an ophthalmologist. His only medications included over the counter analgesics such as acetaminophen and non-steroidal anti-inflammatory drugs for the management of myalgia related pain. No known drug allergies were reported.

Upon examination, the patient weighed 69 kg, measured 190 cm in height, with a calculated BMI of 19.1. He exhibited phenotypical features of Marfan’s Syndrome, including a long thin frame, arachnodactyly, a high arched palate and sternal excavation. He had a normal spinal column alignment, with no evidence of scoliosis or kyphosis. There was no obvious joint contractures or evidence of muscular atrophy. His cardiovascular and respiratory examinations were normal. Upon assessment of his airway, his mouth opening measured over 5 cm, his thyromental distance exceeded 6.5 cm and he had a Mallampati score of 1. A high-arched palate was noted. No restrictions were present in either neck flexion or extension. Vital signs including his blood pressure, heart rate and oxygen saturation levels were within normal ranges. Electrocardiogram and blood results were also normal.

The patient was admitted to hospital the day of his surgery. Intravenous access was established in the forearm avoiding potential contracture sites. Monitoring involved the recording of electrocardiograph, blood pressure, pulse oximetry, end tidal CO2 and temperature. Preoxygenation was administered, followed by an anaesthetic induction consisting of Fentanyl, Propofol and Rocuronium. A depolarising muscle relaxant was avoided. Bag-valve-mask ventilation of the patient was easy achieved without the requirement of an oropharyngeal airway. Laryngoscopy using a McGrath video laryngoscope (size 4 blade) provided a Cormac Lehane grade 1 view for intubation, assisted by using a bougie. A size 8 RAE tube was inserted. Ventilation was delivered without issue using a volume control setting, with the patient receiving 500mls of tidal volume, a peep of 5cmh20, while maintaining peak airway pressures below 19. Sevoflurane at a MAC of 1.2 was used to maintain anaesthesia.

Careful attention was given to patient positioning to prevent pressure sores and nerve entrapment. Throughout the 55-minute surgical procedure, the patient remained haemodynamically. Muscle paralysis was reversed with 200 mg of Suggammadex and confirmed using neuromuscular monitoring, revealing a train of four count of 4. Extubation proceeded without complications. The postoperative course was uneventful, with no evidence of post-operative respiratory depression or compromise, leading to the patients discharge from hospital on the first postoperative day.

## Discussion and conclusions

Bethlem Myopathy, a rare collagen VI-related myopathy, can present a spectrum of clinical challenges for the Anaesthesiologist. Typically presenting in childhood or adolescence as a benign limb girdle myopathy, the disorder is characterised by progressive proximal muscle weakness, peripheral joint laxity and joint contractures [5]. Disease progression can result in patients becoming wheelchair-bound after the age of 50, and respiratory failure may ensue due to progressive respiratory muscle weakness. This decline in respiratory function is variable and occurs later in adulthood, typically after 40 years of age, with some requiring the initiation of nocturnal non-invasive ventiltion [6]. In contrast, Ullrich myopathy, also a collagen VI- related myopathy, manifests at an earlier age and is characterised by a more pronounced phenotypic severity. Affected individuals may never achieve the ability to walk or may lose their ambulatory capacity by the age of 10. Furthermore, pulmonary function deteriorates early on, with the average age for the initiation of non-invasive ventilation being 11 years of age [6].

Despite the relative benign course and slow progression of Bethlem Myopathy when compared to the significantly more severe myopathies such as Duchenne’s muscular dystrophy and Ullrich Myopathy, unique pathophysiological characteristics can present challenges for the anaesthetist. There is limited available literature acknowledging specific anaesthetic considerations for Bethlem myopathy.

From an airway perspective anticipation of a difficult airway is warranted due to the presence of high arched palates, micrognathia and potential fixed flexion deformities of the neck and the possibility of congenital torticollis with this disorder [7].

Although cardiovascular features exist with this disorder, it is important to recognise that it is likely a secondary consequence of the progressive restrictive respiratory disease imposed by Bethlem Myopathy, rather than a direct cardiac involvement of its clinical spectrum [8]. Associated cardiovascular implications include right sided heart failure, right ventricular dysfunction and pulmonary hypertension. Appropriate pre-operative investigations such as an echocardiogram, pulmonary function tests and blood gas analysis may be warranted to establish baseline functionality and to guide anaesthetic management. There are no direct links or reports of an association of malignant hyperthermia in patients with Bethlem Myopathy [9]. Succinylcholine is contra-indicated in myopathies, due to the risk of severe hyperkalaemia, secondary to the upregulation of extra-junctional acetylcholine-receptors [10] .

In cases of prolonged procedures or patients with known respiratory disease, intra-operative monitoring, such as intra-arterial catheters and advanced haemodynamic monitoring should be considered. Due to the inherent risk of a complicated post-operative course, ambulatory surgery is generally not advised in these patients.

Venous access can prove difficult due to the presence of joint contractures. Close attention is required when positioning a patient to prevent pressure sores and nerve entrapment.

Dependent on the degree of existing muscle weakness and the extent of the surgical procedure, post-operative mechanical ventilation may be required.

Bethlem Myopathy, while often exhibiting a mild clinical course, can present distinct anaesthetic challenges, particularly as patients progress into later adulthood due to the disease’s progressive nature and variable clinical manifestations. Awareness of potential complications including a difficult airway, cardiovascular and respiratory implications as well as the need for specialised monitoring and positioning is crucial to ensure a safe peri-operative course.

## Data Availability

No datasets were generated or analysed during the current study.
